# Urinary tract infections in pregnancy in a rural population of Bangladesh: population-based prevalence, risk factors, etiology, and antibiotic resistance

**DOI:** 10.1186/s12884-019-2665-0

**Published:** 2019-12-31

**Authors:** Anne CC Lee, Luke C. Mullany, Alain K. Koffi, Iftekhar Rafiqullah, Rasheda Khanam, Lian V. Folger, Mahmoodur Rahman, Dipak K. Mitra, Alain Labrique, Parul Christian, Jamal Uddin, Parvez Ahmed, Salahuddin Ahmed, Arif Mahmud, Sushil K. DasGupta, Nazma Begum, Mohammad A. Quaiyum, Samir K. Saha, Abdullah H. Baqui

**Affiliations:** 1 Department of Pediatric Newborn Medicine, Brigham and Women’s Hospital, 75 Francis Street, Boston, MA 02115 USA; 2 Department of International Health, Johns Hopkins Bloomberg School of Public Health, 615 N Wolfe St, Baltimore, MD 21205 USA; 3 Department of Microbiology and Immunology, University of Mississippi Medical Center (UMMC), 2500 N State St, Jackson, MS 39216 USA; 4 Maternal and Child Health Division, International Centre for Diarrhoeal Disease Research, Bangladesh (icddr,b), 68, Shaheed Tajuddin Ahmed Sarani, Mohakhali, Dhaka, 1212 Bangladesh; 5 North South University, Plot #15, Block #B, Bashundhara R/A, Dhaka, 1229 Bangladesh; 6 Bill and Melinda Gates Foundation, 440 5th Avenue North, Seattle, WA 98109 USA; 7 Save the Children Bangladesh, House No. CWN (A) 35, Road No. 43 Gulshan 2, Dhaka, 1212 Bangladesh; 8 Institute of Epidemiology Disease Control and Research, Mohakhali, Dhaka, 1212 Bangladesh; 9 Projahnmo Research Foundation, House: 37, Road:27, Block: A, Banani, Dhaka, 1213 Bangladesh; 10 School of Community Health and Policy, Morgan State University, Baltimore, MD 21251 USA; 11 Department of Microbiology, Dhaka Shishu Hospital, Sher-E-Banglanagar, Child Health Research Foundation, Dhaka, 1207 Bangladesh

**Keywords:** Urinary tract infection, Asymptomatic bacteriuria, Bangladesh, Pregnancy, Maternal morbidity, Risk factors, Antimicrobial resistance

## Abstract

**Background:**

Urinary tract infection (UTI) in pregnancy, including asymptomatic bacteriuria, is associated with maternal morbidity and adverse pregnancy outcomes, including preterm birth and low birthweight. In low-middle income countries (LMICs), the capacity for screening and treatment of UTIs is limited. The objective of this study was to describe the population-based prevalence, risk factors, etiology and antimicrobial resistance patterns of UTIs in pregnancy in Bangladesh.

**Methods:**

In a community-based cohort in Sylhet district, Bangladesh, urine specimens were collected at the household level in 4242 pregnant women (< 20 weeks gestation) for culture and antibiotic susceptibility testing. Basic descriptive analysis was performed, as well as logistic regression to calculate adjusted odds ratios (aOR) for UTI risk factors.

**Results:**

The prevalence of UTI was 8.9% (4.4% symptomatic UTI, 4.5% asymptomatic bacteriuria). Risk factors for UTI in this population included maternal undernutrition (mid-upper arm circumference <23 cm: aOR= 1.29, 95% CI: 1.03–1.61), primiparity (aOR= 1.45, 95% CI: 1.15–1.84), and low paternal education (no education: aOR= 1.56, 95% CI: 1.09–2.22). The predominant uro-pathogens were *E. coli* (38% of isolates)*, Klebsiella* (12%), and staphyloccocal species (23%). Group B streptococcus accounted for 5.3% of uro-pathogens. Rates of antibiotic resistance were high, with only two-thirds of *E. coli* susceptible to 3^rd^ generation cephalosporins.

**Conclusions:**

In Sylhet, Bangladesh, one in 11 women had a UTI in pregnancy, and approximately half of cases were asymptomatic. There is a need for low-cost and accurate methods for UTI screening in pregnancy and efforts to address increasing rates of antibiotic resistance in LMIC.

## Background

Urinary tract infections (UTI) in pregnancy are a large and under-emphasized risk factor for pregnancy morbidity and adverse birth outcomes in low- and middle-income country (LMIC) settings [1]. UTI may present in pregnancy with symptoms of acute cystitis or pyelonephritis, or may be more insidious in women with asymptomatic bacteriuria (ASB). Screening and treatment of ASB by urine culture is recommended for all women at least once in early pregnancy in high-income countries, by the Infectious Diseases Society of America [2], Canadian Task Force on Preventive Care [3], and National Institute of Health and Clinical Excellence of the United Kingdom (UK) [4]. In low-income countries, screening and treatment of UTI or ASB is challenging due to the costs and logistics of performing urine culture. Recently, the World Health Organization (WHO) made context-specific antenatal care recommendations for screening and treatment of ASB in LMIC [5], recommending urine culture in settings with capacity, or mid-stream urine Gram stain, and treatment of ASB.

There is a paucity of population-based data on the prevalence and etiology of UTI in pregnancy in low-middle income countries. In a recent review, the global prevalence of UTI and/or ASB in pregnancy ranged from 3 to 35% across 5 continents in countries with preterm birth rates > 10% [1]. Women carry higher risk of UTI than men, and pregnancy places women at increased risk of ascending infection due to the weight of the fetus and dilation of the ureters and renal pelvis [6, 7]. Before urine culture was standard of care in the US (1960’s), pyelonephritis developed in 40% of pregnant women with untreated bacteriuria [8]. Maternal urinary tract infections may trigger an inflammatory response, including the release of chemokines and cytokines that may result in decidual activation, prostaglandin release, and cervical ripening, thereby increasing the risk of preterm birth [9]. In historical studies, approximately 30–50% of women with pyelonephritis delivered preterm [10–12]. ASB is significantly associated with preterm delivery (RR 2·00, 95% CI 1·43–2·77) [13], and low birthweight (RR 1.54, 95% CI 1.35–1.75); however, evidence for the impact of ASB screening and treatment on preterm birth risk has been graded as weak [14]. In addition, maternal UTI has been associated with increased risk of stillbirth [15] and early onset neonatal sepsis [16].

We recently screened pregnant women for UTI as part of a cluster-randomized controlled trial (clinicaltrials.gov identifier: NCT01572532) designed to evaluate the impact of a community-based antenatal screening and treatment program for genito-urinary tract infections in early pregnancy on population-level rates of preterm birth in rural Sylhet district, Bangladesh [17, 18]. In this manuscript, we describe the population-based prevalence, risk factors, etiology, and antimicrobial resistance patterns of UTIs in this cohort.

## Methods

### Study population and study design

The data reported in this manuscript were part of a population-based cluster randomized controlled trial (cRCT) to determine the impact of an antenatal screening program for abnormal vaginal flora and UTI on the incidence of preterm live birth (clinicaltrials.gov identifier: NCT01572532) [14, 15]. The study protocol was approved by the institutional review boards of Johns Hopkins Bloomberg School of Public Health Institutional Review Board (Baltimore, Maryland), International Centre for Diarrhoeal Disease Research, Bangladesh Ethical Review Committee (iccdr,b) (Dhaka, Bangladesh), and Partners Human Research Committee (Boston, MA). The Maternal Infection Screening and Treatment (MIST) study [17] was conducted between January 2, 2012 and July 28, 2015 in two rural sub-districts of Sylhet district, Bangladesh: Zakiganj and Khanaighat, which are part of the Projahnmo research site. The study site is in a rural, agrarian area, with poor access to health care and high need, having one of the highest neonatal mortality rates in Bangladesh. Health services in Bangladesh are provided by the government’s Ministry of Health and Family Welfare, NGOs, and private providers. According to the 2014 Bangladesh Demographic and Health Survey, 79% of pregnant women had at least one ANC visit and 31% received at least four ANC visits [19]. Screening for urinary tract infection in pregnancy by urine culture is not standard of care in Bangladesh.

A cluster was defined as the area served by a community health worker (CHW), comprising several contiguous villages (population~ 4000 people, approximately 120 annual pregnancies, per cluster). In MIST, there were 24 clusters, for which community health workers conducted home visits to provide basic antenatal and post-partum care and education. In the cRCT, 12 clusters were randomized to receive the intervention, which included home-based screening and treatment of UTI and abnormal vaginal flora (AVF). In this manuscript, we report the results of the UTI screening. Results of the AVF intervention are reported elsewhere [20].

### Pregnancy surveillance

All married women of reproductive age in the study areas were under routine monthly community-based pregnancy surveillance through the duration of the study. Pregnant women who had no or uncertain recall of their last menstrual period (LMP), LMP > 19 weeks, self-reported history of irregular bleeding due to injectable depoprovera, or self-reported history of severe chronic disease were excluded. Participants were enrolled after providing verbal informed consent. Verbal consent was obtained because the majority of the prospective study population was illiterate at the initiation of the study. Documentation of verbal informed consent was formally approved by all institutional review boards.

### Urinary tract infection screening

For pregnant women in intervention clusters, during a home visit at < 19 weeks gestation, a clean catch midstream urine specimen was collected for culture. The CHW instructed the mother to spread the labia widely before collecting 20-30 mL of the midstream urine into a sterile wide-mouthed container. The urine specimen was immediately placed in a cooler refrigerated with ice-packs (~ 2 to 8 °C) and transported to the Sylhet field laboratory, maintaining the cold chain. A random selection of 10% of the control arm women also had urine screened by culture.

### Laboratory testing

Urine specimens were inoculated on standard MacConkey and Blood agar plates within 6 h of collection and incubated at 37 **°**C for 48 h. Bacterial growth was quantified twice (24 and 48 h). Bacterial isolates were speciated using standard microbial techniques. Antibiotic susceptibility testing was performed on Mueller-Hinton agar plates following standard laboratory protocols. The disk diffusion method was used; zones of growth inhibition were measured in mm [21]. The antimicrobial discs used were: cefixime, nitrofurantoin, ampicillin, azithromycin, cotrimoxazole, gentamycin, nalidixic acid, ceftriaxone, and cephalexin.

### Definitions

We defined categories of urinary tract infection shown in Table 1. The list of organisms that we considered as UTI pathogens versus contaminants is shown in Additional file 1: Table S1.

**Table 1:** Clinical Categories of Urinary Tract Infection, Based on Culture Growth and Symptoms

UTI Terminology	Definition
*High-burden growth*	bacteriuria of > 10^5^ colony forming units (CFU) per 1 mL of urine of a single uropathogen [22]
*Intermediate growth*	bacteriuria with > 10^3^ -10^5^ CFU/mL of a single uropathogen
*Contamination*	bacterial growth of > 1 micro-organism OR growth of a non-urinary tract pathogen (Additional file 1: Table S1)
*UTI symptoms*	dysuria, urinary frequency, hematuria, abdominal pain, fever, OR flank pain
*Symptomatic intermediate growth*	women with intermediate burden growth and UTI symptoms (as above)
*Asymptomatic Bacteriuria*	women with high burden bacterial growth without UTI symptoms
*Cystitis*	women with positive urine culture (high burden or intermediate growth) and symptoms of dysuria, urinary frequency, hematuria, urinary urgency or suprapubic tenderness, without upper urinary tract symptoms (fever, chills, flank or back pain) [22]
*Pyelonephritis*	women with positive urine culture and systemic symptoms (fever, chills, flank pain or back pain) [22]

### UTI treatment

The MIST treatment algorithm for positive urine cultures is shown in Additional file 1: Figure S1. UTI was treated in pregnant women with high burden growth (> 10^5^ CFU/mL of a uro-pathogen), as well as among women with intermediate growth who had UTI symptoms, as per ACOG recommendations [22]. From January to October 2012, the first line antibiotic treatment was cefixime 400 mg po once daily for 3 days. After observing that only two-thirds of *E. coli* were susceptible to cefixime, the first-line treatment was then changed to nitrofurantoin (Macrobid) 100 mg po bid for 7 days. All symptomatic women were also referred to the sub-district hospital for further evaluation and management.

A test of cure was obtained 1 week after antibiotic completion for all pregnant women with positive cultures. If the second urine culture remained positive, the supervising field physician prescribed an antibiotic based on the antimicrobial sensitivity pattern. Persistent UTI after twice-repeated treatment was referred to Sylhet Osmani Medical College Hospital for further evaluation and management.

### Data analysis

Simple descriptive statistics were used to describe the distribution of uro-pathogens and antibiotic sensitivity patterns.

For the risk factor analysis, the primary outcome was UTI requiring antibiotic treatment, as defined in Table 1. We included a number of socio-demographic characteristics, nutrition, behavior, and reproductive history variables as potential risk factors. Socio-demographic characteristics included household wealth, household size, religion, woman’s age at enrollment, parity, and educational attainment of both the woman and her husband. All variables were categorized. A wealth index indicative of relative economic status of the household within the study population was constructed using household facilities and assets weighted with principal component analysis [23]; the households were categorized into quintiles. Comparisons of proportions between groups were analyzed by chi-squared statistic.

Risk factors with *p* < 0.05 in bivariate analysis were included in multivariate analysis. Logistic regression was performed to calculate adjusted odds ratios (aORs) and 95% confidence intervals using STATA version 14.0 (StataCorp LP, College Station, TX).

## Results

**Fig. 1:**
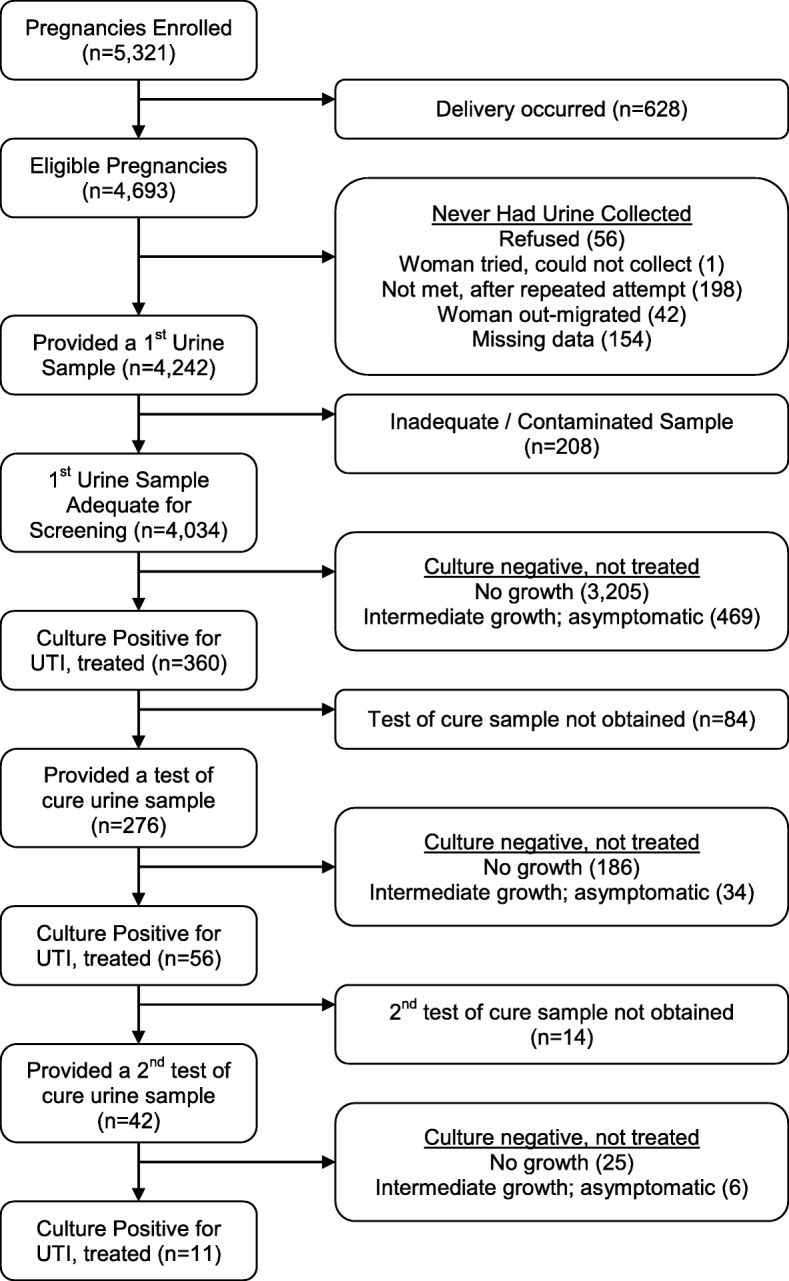
CONSORT Flow Diagram for MIST Study UTI Samples

Figure 1 shows a flow chart of patient enrollment and specimens collected as part of the study intervention. The overall rate of urine culture contamination was 4.9% (208/4242). These specimens were excluded from the results reported in Table 2, which report on the first adequate, non-contaminated urine specimens (*n* = 4034).

**Table 2:** Prevalence of UTI

	Number	Percent	95% CI (%)
First adequate screening specimen^a^	4034		
> 100,000 CFU/mL, symptomatic	47	1.2	0.9–1.5
> 100,000 CFU/mL, asymptomatic	183	4.5	3.9–5.2
1000- < 100,000 CFU/mL, symptomatic	130	3.2	2.7–3.8
1000- < 100,000 CFU/mL, asymptomatic	469	11.6	10.7–12.6
No Growth (Normal)	3205	79.5	78.2–80.7
First Test of cure specimen	276		
> 100,000 CFU/mL, symptomatic	5	1.8	0.8–4.3
> 100,000 CFU/mL, asymptomatic	36	13.0	9.6–17.7
1000- < 100,000 CFU/mL, symptomatic	15	5.4	3.3–8.9
1000- < 100,000 CFU/mL, asymptomatic	34	12.3	9.0–16.9
No Growth (Normal)	186	67.4	62.1–73.2
Second Test of cure specimen	42		
> 100,000 CFU/mL, symptomatic	2	4.8	1.2–18.4
> 100,000 CFU/mL, asymptomatic	8	19.0	10.2–35.5
1000- < 100,000 CFU/mL, symptomatic	1	2.4	0.3–16.5
1000- < 100,000 CFU/mL, asymptomatic	6	14.3	6.8–30.0
No Growth (Normal)	25	59.5	46.4–76.4

^a^This table presents by order of adequate specimen. That is, some specimens listed under “1st specimen”, include a small number of specimens that were in fact second specimen collected (but represented first testable/non-contaminated specimen)

### Prevalence of UTI

In the initial screening of urine samples from the general pregnancy population (n = 4034), 230 (5.7%) women had high burden (> 100,000 CFU/mL) bacteriuria, among which 47 (20.4%) reported clinical signs of UTI, and 183 (79.6%) had no symptoms (Table 2). An additional 599 (14.9%) women in the initial screening had intermediate level (> 1000- < 100,000 CFU/mL) bacteriuria, with 130 (21.7%) of those women reporting UTI symptoms, which were treated with antibiotics. Overall, 360 (8.9%) of women required treatment for UTI, of whom 308 (86%) were started on antibiotics and 284 (92%) completed the full course.

Among women with positive screening urine cultures, we successfully obtained a test of cure specimen in 276/360 women. Among these mothers, 56/276 (20.3%) had a persistent UTI on the second urine culture that required a second course of antibiotics, that was selected based on antimicrobial resistance patterns. Among these women, we obtained a final test of cure specimen from 42 women with persistent UTI. The prevalence of persistent UTI among those women was 26.2% on the final screening.

### Risk factors for UTI

Table 3 shows the analysis of risk factors for UTI in this population. In bivariate analysis, low household wealth, primiparity, and maternal undernutrition as measured by MUAC were associated with increased risk of UTI. Higher education of the women and their husbands were protective for UTI. In multivariate analysis (Table 4), low MUAC, low paternal education, and primiparity remained statistically significant risk factors for UTI.

**Table 3:** Distribution of potential risk factors by UTI status, bivariate analysis

	Total	No UTI (*N* = 3674)	UTI (N = 360)	χ^2^ (*p*-value)
Socio-demographic characteristic				
Household wealth quintiles (missing: 1)				
Lowest tertile	1361	1217 (89.4%)	144 (10.6%)	7.867 (0.0196)
Middle tertile	1335	1220 (91.4%)	115 (8.6%)	
Highest tertile	1338	1237 (92.5%)	101 (7.6%)	
Hand washing station (missing; 0)				
No	3924	3575 (91.1%)	349 (8.9%)	0.161 (0.688)
Yes	110	99 (90.0%)	11 (10.0%)	
Household size (missing: 1)				
1–3 household members	649	584 (90.0%)	65 (10.0%)	2.529 (0.283)
4–6 household members	1730	1589 (91.9%)	141 (8.1%)	
7+ household members	1654	1500 (90.7%)	154 (9.3%)	
Low MUAC at enrollment (missing: 8)				
No	2419	2228 (92.1%)	191 (7.9%)	7.782 (0.005)
Yes	1607	1439 (89.6%)	168 (10.4%)	
Religion (missing: 1)				
Islam	3871	3527 (91.1%)	344 (8.9%)	0.187 (0.665)
Other	162	146 (90.1%)	16 (9.9%)	
Mother’s age at enrollment (missing: 0)				
< 20	381	341 (89.5%)	40 (10.5%)	2.044 (0.360)
20–29	2567	2349 (91.5%)	218 (8.5%)	
30 or more	1086	984 90.6%)	102 (9.4%)	
Mother’s education (missing: 0)				
No Education (0 years)	786	695 (88.4%)	91 (11.6%)	10.280 (0.0059)
Primary Education (1–5 years)	1522	1385 (91.0%)	137 (9.0%)	
Secondary or higher Education (6+)	1726	1594 (92.4%)	132 (7.6%)	
Husband’s education (missing: 0)				
No Education (0 years)	1360	1213 (89.2%)	147 (10.8%)	15.665 (0.0004)
Primary Education (1–5 years)	1572	1428 (90.8%)	144 (9.2%)	
Secondary or higher Education (6+ years)	1102	1033 (93.7%)	69 (6.3%)	
Reproductive and sexual history				
Primiparous at enrollment (missing: 1)				
No	2710	2486 (91.7%)	224 (8.3%)	4.436 (0.035)
Yes	1323	1187 (89.7%)	136 (10.3%)	
Hormonal contraceptive history (missing: 0)				
No	3964	3611 (91.1%)	353 (8.9%)	0.101 (0.7501)
Yes	70	63 (90.0%)	7 (10.0%)

**Table 4:** UTI Risk Factors, Multivariate Analysis

	Odds Ratio	95% Confidence Interval
Household wealth tertiles		
Lowest tertile	1.05	0.75–1.48
Middle tertile	0.96	0.71–1.30
Highest tertile	1.00	–
Low MUAC (< 23 cm) at enrollment		
No	1.00	–
Yes	1.29	1.03–1.61
Mother’s education		
No Education (0 years)	1.36	0.94–1.96
Primary Education (1–5 years)	1.11	0.85–1.47
Secondary or higher Education (6+)	1.00	–
Husband’s education		
No Education (0 years)	1.56	1.09–2.22
Primary Education (1–5 years)	1.42	1.04–1.94
Secondary or higher Education (6+ years)	1.00	–
Primiparous at enrollment		
No	1.00	–
Yes	1.45	1.15–1.84

### Etiology of UTI

**Fig. 2:**
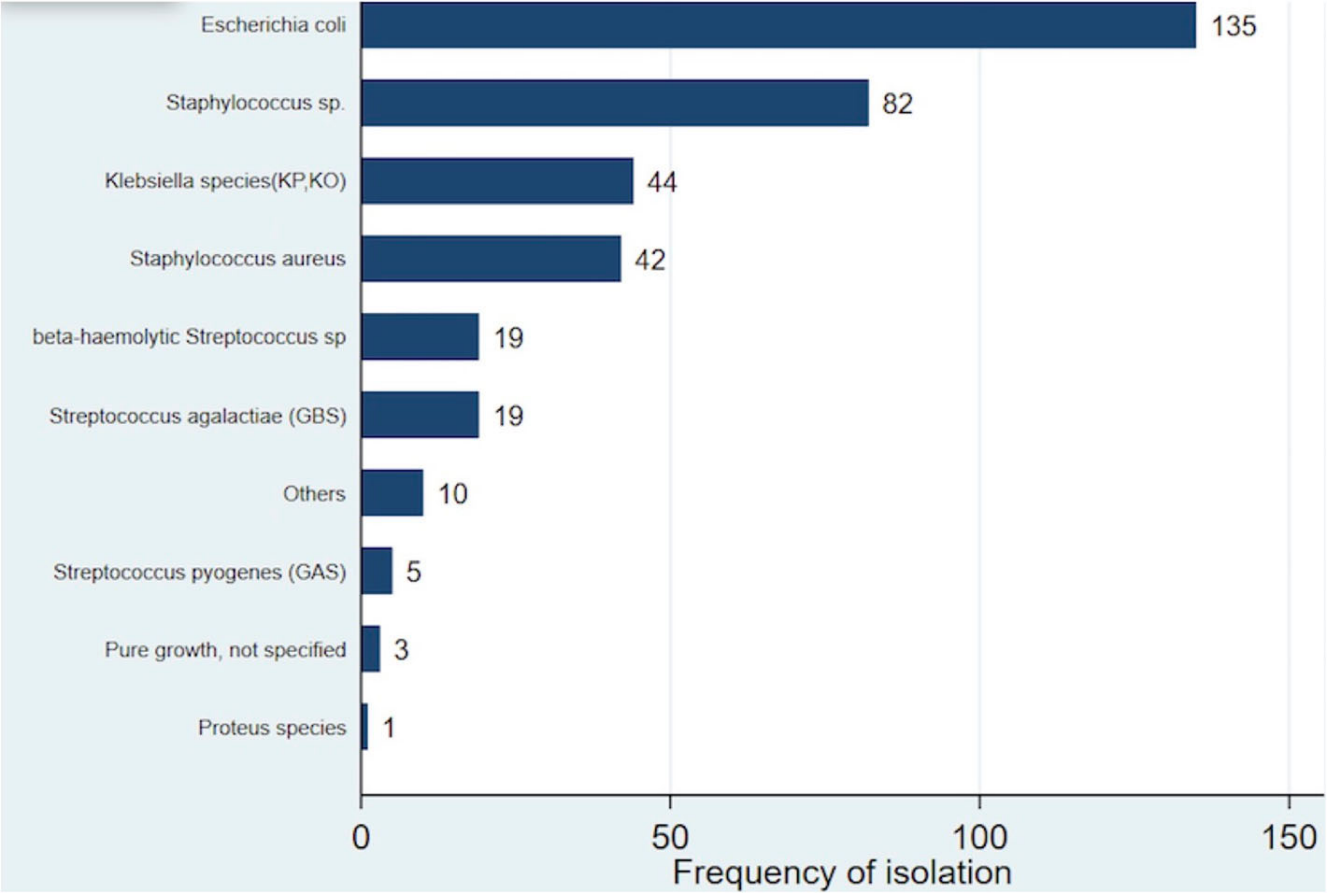
UTI Pathogens in First Adequate Urine Screening

Figure 2 shows the bacterial etiology of UTI pathogens among pregnant mothers who were treated (high burden and intermediate growth in symptomatic mothers; *n* = 360) in the initial screening of the general pregnancy population in Sylhet, Bangladesh. The predominant micro-organisms were *E. coli* (*n* = 135; 38% of isolates) and *Staphylococcus* species (non-aureus) (*n* = 82; 23%), followed by *Klebsiella* species (*n* = 44, 12%). *Staphylococcus aureus* was common, isolated in 42 (12%) women with UTI. The prevalence of Group B *Streptococcus* (GBS) bacteriuria was generally lower, with 19 isolates (*n* = 19; 5.3%).

Among the intermediate growth in mothers who were asymptomatic, there was a predominance of skin contaminants, including staph and strep species, and these are shown in the Additional file 1: Figure S2.

### Antimicrobial susceptibility patterns

Table 5 shows the antimicrobial susceptibility patterns of uro-pathogens in the MIST study. *E. coli* isolates had low rates of susceptibility to ampicillin (34% of isolates) and azithromycin (28%); susceptibility to cefixime, cotrimoxazole, and cephalexin were in the moderate range (69, 63, and 62% respectively). The majority of species were highly susceptible to nitrofurantoin, with the exception of *Klebsiella* species, where 74% of strains were susceptible. Rates of susceptibility to azithromycin were low among the gram-negative species.

**Table 5:** Antibiotic Susceptibility Patterns of Uropathogens in the MIST Study

	*Escherichia coli*	*Klebsiella species*	*Staphylococcus aureus*	*Staph species (non-aureus)*	*Group B Strep*	*Beta-hemolytic Streptococcus*
Cefixime	82/119^a^ (68.9%)	34/36 (94.4%)	0/7	4/28 (14.3%)	4/7 (57.1%)	11/11 (100.0%)
Nitrofurantoin	117/118 (99.2%)	26/35 (74.3%)	7/7 (100.0%)	28/28 (100.0%)	7/7 (100.0%)	11/11 (100.0%)
Ampicillin	40/119 (33.6%)	3/36 (8.3%)	3/7 (42.9%)	16/28 (57.1%)	7/7 (100.0%)	10/11 (90.9%)
Azithromycin	34/120 (28.3%)	3/36 (8.3%)	3/7 (42.9%)	22/28 (78.6%)	2/7 (28.6%)	6/11 (54.5%)
Cotrimoxazole	74/118 (62.7%)	35/36 (97.2%)	6/7 (85.7%)	23.28 (82.1%)	7/7 (100.0%)	11/11 (100.0%)
Gentamicin	97/117 (82.9%)	33/36 (91.7%)	6/7 (85.7%)	24/28 (85.7%)	3/7 (42.9%)	2/7 (18.2%)
Nalidixic acid	45/121 (37.2%)	33/36 (91.7%)	0/7 (0.0%)	5/28 (17.9%)	0/7 (0.0%)	0/11 (0.0%)
Cetriaxone	83/117 (70.9%)	31/35 (88.6%)	5/7 (71.4%)	24/25 (96.0%)	7/7 (100.0%)	11/11 (100.0%)
Cephalexin	74/119 (62.2%)	29/36 (80.6%)	6/7 (85.7%)	26/28 (92.9%)	5/7 (71.4%)	11/711 (100.0%)

^a^n/N in each cell report the (number of bacterial isolates susceptible to the specified antibiotic)/(number of urine samples with bacterial species isolated)

## Discussion

In a cohort of pregnant women in rural Sylhet, Bangladesh, the prevalence of UTI in early pregnancy was 8.9% (4.4% symptomatic UTI, 4.5% asymptomatic bacteriuria). A majority of women with bacteriuria in pregnancy were asymptomatic. Risk factors for UTI in this population included maternal undernutrition, primiparity, and low paternal education. The common uro-pathogens were similar to those reported in other geographies, with a predominance of gram negatives, including *E. coli* and *Klebsiella*, as well as staphyloccocal species. Group B streptococcus accounted for only 5.3% of uro-pathogens. Rates of antibiotic resistance were high, with greater than 30% of *E. coli* resistant to 3rd generation cephalosporins.

The prevalence of UTI/ASB in our study population was comparable to other studies in South Asia. In our study, we sampled all pregnant women identified from households in the study catchment area. This differs from the majority of studies, which recruited pregnant women presenting at ANC clinics or tertiary care facilities. One Bangladeshi study recruiting from ANC clinics reported a 5% bacteriuria rate, with 1% of women presenting with UTI symptoms [24]. Reports from urban and rural Rajshahi district, Bangladesh reported that 4–12% of women presenting to antenatal care had asymptomatic bacteriuria [25, 26]. In a study of mothers at an ANC clinic in rural Nagpur, India the culture-positive UTI (symptomatic and asymptomatic) prevalence was 9.6%, similar to our study [27]. Two studies in urban settings in northern India reported higher prevalence of ASB and UTI, ranging from 19.9% ASB prevalence in primary care clinics [28] to 25.5% prevalence of symptomatic UTI in a tertiary care ANC clinic in Lucknow [29].

In our pregnancy cohort, the majority of women with bacteriuria in were asymptomatic. This has relevance with respect to screening procedures in LMIC. A symptomatic approach to UTI will miss the majority of cases and the opportunity for intervention-treatment to prevent maternal morbidity and adverse pregnancy outcomes. While urine culture is standard of care in high income countries (HIC), it is typically costly and requires laboratory resources, infrastructure, and personnel and is not feasible in many LMIC settings. The diagnostic accuracy of urine dipstick and gram stain for diagnosis of ASB is poor, with particularly low sensitivity [30, 31]. Lower cost, feasible, and accurate point of care methods/diagnostics for screening for ASB are urgently needed to improve detection and management of UTI in LMIC.

Primiparity, less paternal education, and maternal undernutrition were significant risk factors for UTI in this population. Poor hygiene practices may be more common in first time mothers of young age and those with low SES, and predispose them to urinary tract infection [32]. Low paternal education is a marker for low SES, a frequently reported risk factor for UTI [32]. Maternal undernutrition, defined as maternal MUAC < 23 cm, was also observed to be a risk factor for UTI in this population. Malnutrition is an important and under-recognized cause of immunodeficiency globally [33]. Protein energy malnutrition may impair immune function (i.e. antigen-presenting cell and cell mediated T-cell function), and increase risk of maternal infections, including UTI [34–36]. Undernutrition has been identified as a risk for UTI in children [37] and the elderly [38]. However, to our knowledge, this is the first report of this association between maternal undernutrition and UTI in a pregnancy population in a LMIC.

Gram-negative organisms, *E. coli* and *Klebsiella* species, were common etiologies of UTI in Sylhet, accounting for half (38 and 12%, respectively) of cases of significant bacteriuria. Other studies of UTI etiology in Bangladesh have similarly reported a predominance of gram negatives, particularly *E. coli,* which comprised 59–75% of isolates, and *Klebsiella* species, which ranged from 6 to 11% of isolates [39, 40]. In a 5-year, large, prospective study of pregnant women in a tertiary care hospital in India, *E. coli* and *Klebsiella pneumoniae* were the most common uro-pathogens (42 and 22% of isolates, respectively) [41].

In this population, there was also a high rate of isolation of gram-positive organisms. Specifically, staphylococcal species (non-aureus) were the second most common uro-pathogen overall, contributing to 23% of positive cultures. The majority of these isolates were presumably *Staphylococcus saprophyticus*, a leading cause of cystitis in young women [42]. However, our field laboratory did not have the capacity to further speciate with novobiocin resistance testing. Other studies in Bangladesh have reported that *S. saprophyticus* [43] comprised 11–19% of uro-pathogens [39, 44]. In India, *S. saprophyticus* comprised 10.6% of positive cultures [45]. While *S. aureus* is often considered skin flora, it accounted for 12% of cases of UTI in Sylhet. While precautions were taken to avoid skin contamination, it is difficult to ascertain whether the bacteriuria was due to skin contamination or whether it was a true uro-pathogen [1]. Among cases of *S. aureus* bacteriuria, approximately 82% were asymptomatic. *S. aureus* was described as an emerging etiology of UTI in LMIC in a recent global burden review [1]. In Nigeria, *S. aureus* comprised approximately 24 to 28% of isolates in women with bacteriuria or clinical UTI [46, 47]. A study of pregnancy-associated ASB in Sudan reported that *S. aureus* comprised 39% of cases [48]. A lower contribution of *S. aureus* infection was reported in India (5.9%) [41].

Antibiotic resistance is a growing concern, particularly in LMIC, and our study demonstrates high and concerning rates of antibiotic resistance to common antimicrobial agents for treatment of UTI in pregnancy. The gram-negative uro-pathogens were highly resistant to ampicillin and azithromycin. More than 30% of *E. coli* isolates were not susceptible to common 2^nd^ and 3^rd^ generation cephalosporins. Among the most common uro-pathogens, *E. coli* and staphylococcal species, there was only low-to- moderate susceptibility to cefixime, a traditionally potent oral 3^rd^ generation cephalosporin. Similar high and concerning rates of antibiotic resistance were reported in the WHO Global Surveillance of Antimicrobials. In national level data from South-East Asia, 16–68% of *E. coli* isolates, and 34–81% of *Klebsiella* isolates were resistant to 3^rd^ generation cephalosporins [49, 50]. This data emphasizes the urgency for antibiotic stewardship in LMIC, and the need to also develop new effective antimicrobials with safety in pregnancy.

There were several limitations to this study. We did not have the ability to speciate coagulase-negative staphylococcal species in our field laboratory. We presume the majority of these species were *Staphylococcus saprophyticus*, however, it is possible that some of these may have been *Staphylococcus epidermidis*, which might be considered a skin contaminant. Another challenge is the differentiation of skin contamination vs. true pathogens. The rates of *S. aureus* growth were high, and it is difficult, if not impossible, to determine what proportion of those should be considered as UTI pathogens, particularly in asymptomatic women. While we used clean catch midstream urine specimens, the samples were collected in homes, and it is possible that some were skin contaminants. We also did not systematically collect cost data, which would have been useful to determine the cost-effectiveness of our program. There is a paucity of evidence on the cost-effectiveness of screening-treatment programs for UTI in pregnancy, which is of particular relevance in LMICs. Finally, we did not have extensive data on several known behavioral risk factors for UTI, including sexual history or toileting practices.

## Conclusions

The screening and treatment of urinary tract infections in pregnancy is standard of care in high-income countries and is now recommended by the WHO for LMIC. In rural Sylhet, Bangladesh, an estimated one in 11 pregnant women in the general population had a UTI that required antibiotic treatment. The majority of women with bacteriuria had no symptoms. Low paternal education, primparity, and maternal undernutrition were important risk factors for UTI. Rates of antibiotic resistance were concerningly high, particularly in *E. coli* strains. Further research is needed to identify low-cost, feasible, and accurate methods for UTI screening and to address high rates of antibiotic resistance in LMIC.

## Supplementary information

**Additional file 1 : Table S1.** Classification of Common Urinary Tract Pathogens and Non-Pathogens. **Figure S1.** MIST treatment algorithm for positive urine cultures. **Figure S2.** Etiology of bacteria among women with intermediate growth cultures who were asymptomatic.

## Data Availability

The datasets used and/or analyzed during the current study are available from the corresponding author on reasonable request.

## Abbreviations

ANC: Antenatal care
ASB: Asymptomatic bacteriuria
AVF: Atypical vaginal flora
CHW: Community health worker
LMICs: Low- and middle-income countries
LMP: Last menstrual period
MIST: Maternal infection screening and treatment (study)
MUAC: Mid-upper arm circumference
UTI: Urinary tract infection
WHO: World Health Organization
