# Supplementary material for: Urinary tract infections in pregnancy in a rural population of Bangladesh: population-based prevalence, risk factors, etiology, and antibiotic resistance
Source: BMC Pregnancy Childbirth. 2019 Dec 31;20:1. doi: 10.1186/s12884-019-2665-0 (PMC6938613; doi:10.1186/s12884-019-2665-0)
Supplement: Supplementary file 1 — Additional file 1 : Table S1. Classification of Common Urinary Tract Pathogens and Non-Pathogens. Figure S1. MIST treatment algorithm for positive urine cultures. Figure S2. Etiology of bacteria among women with intermediate growth cultures who were asymptomatic. [file 12884_2019_2665_MOESM1_ESM.docx]

**Supplemental Table 1.** Classification of Common Urinary Tract Pathogens and Non-Pathogens

| Common Urinary Tract Pathogens | Uncommon Urinary Tract Pathogen | Non-Pathogens |
| --- | --- | --- |
| *E Coli* | Acinetobacter | Lactobacillus |
| Klebsiella species | Pseudomonas | Corynebacterium |
| Proteus species | Providencia | Coag negative Staph |
| Enterobacteriaceae |  | Alpha hemolytic strep |
| Enterococcus faecalis |  | Nonhemolytic strep |
| Staph aureus |  |  |
| Staph saphrophyticus |  |  |
| Beta haemolytic streptococcus |  |  |

**Supplemental Figure 1.** MIST treatment algorithm for positive urine cultures


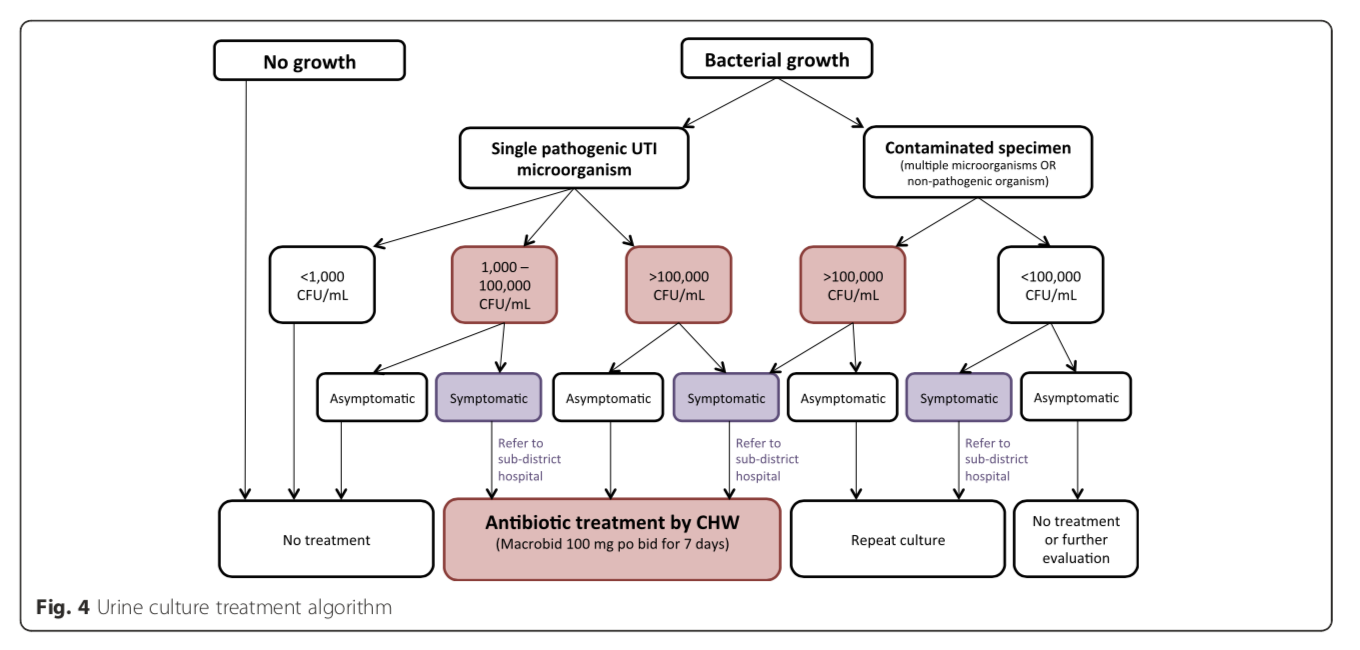


**Supplemental Figure 2.** Etiology of bacteria among women with intermediate growth cultures who were asymptomatic
